# Blazing a Trail: A Public Health Research Agenda in Genomics and Chronic Disease

**Published:** 2005-03-15

**Authors:** Colleen M McBride

**Affiliations:** National Human Genome Research Institute

Whether and when genomics will lead to public health benefit via reductions in chronic disease burden has provided fodder for debate (1,2). A point of agreement among both proponents and skeptics is that directing genomics research to achieve this end will require integration of knowledge across multiple disciplines and levels of analysis (i.e., biological, behavioral, social, and environmental) (3). Getting started on building these collaborations while the territory is new could temper the disciplinary hegemony that so often presents formidable barriers to transdisciplinary research (4). That said, when it comes to genomics, which has been the bastion of bench scientists and most recently epidemiologists, it may be especially challenging to attract the array of chronic disease researchers with expertise in health education, health psychology, health services delivery, and community-based intervention that will be critical to further this research agenda.

Vociferous pessimism expressed by some scientific leaders about the future application of genomic discovery to public health improvements (2) may be scaring off some public health scientists from pursuing genomics research (5). As has been said, "mud sticks whatever its veracity" (6). However, some public health researchers (7) would contend that waiting until genomic discovery is further along to get involved will relegate us to the role of translators, stuck with disseminating the technologies that evolve, even if they are poorly suited to populations or limited in their impact on chronic disease outcomes. Indeed, public health scientists must be among the trailblazers in step with or a step ahead of the science, with a voice in directing genomics research toward public health benefit.

Unfortunately, the emerging public health research agenda for chronic disease is giving relatively little consideration to the future of genomic discovery (8). An informal review of the *American Journal of Public Health* over the past decade shows that from 1995 to 1999 only eight articles related to genetics were published, a number that increased only to 22 between 2000 and 2004. Publications related to obesity, another area recognized during the same time to be important for chronic disease, increased fivefold from 26 to 138.

So how do we enlist public health scientists in transdisciplinary collaborations that further a public health research agenda? First, it is time for a frame change. The past decade's research agenda was framed to anticipate and protect the public from the potential negative ethical, social, and psychological implications of genomic discovery. Not surprisingly, scientists in the vanguard of this research have been bioethicists, lawyers, and public policy experts. To enlist public health researchers in genomics research, the agenda must be reframed to understand the practical and proximal benefits of genomics for chronic disease. Specifically, we should be figuring out how genomic discovery might help us to address three persistent challenges for chronic disease prevention and management: 1) reducing prevalent behavioral risk factors, 2) reducing disparities in chronic disease outcomes, and 3) improving chronic disease care delivery at reduced cost. Below I suggest examples of research in genomics and chronic disease that could galvanize the transdisciplinary research collaborations needed to address these challenges.

## Reducing prevalent behavioral risk factors

The predicted broad array of genetic susceptibility tests that will identify populations and individuals at increased risk of chronic disease raise myriad research questions. Most notably, how can these tests and related feedback be used to motivate adoption of risk-reducing behaviors?

At the social environment level, public education about genomics will be a priority. Fewer than half of Americans are aware of currently available genetic testing for cancer susceptibility (9). Not surprisingly, awareness is greatest among the most highly educated. Contrast this to a recent Institute of Medicine report suggesting that nearly half of Americans cannot read complex text and may lack the skills needed to evaluate the risks and benefits of health-related technologies (10). Development and evaluation of health education approaches for individuals with low literacy is needed generally, and testing strategies to communicate the complexity of genomic risk may be especially fertile ground for this research. The increasing direct-to-consumer advertising of susceptibility testing and popular press coverage of genomic discovery provide a number of "interventions" and natural experiment opportunities for exploring the public's understanding of genomic risk and examining factors that influence interest in testing and its association with risk-reduction outcomes across different target groups.

At an individual level, a number of social and psychological theories support debate about whether genomic risk information will be viewed as more motivational for risk-factor reduction than other risk feedback (e.g., measurement of blood pressure and cholesterol levels, family history). Important questions remain about whether genetic risk information can help us improve upon state-of-the-art risk communications by personalizing risk in different or more effective ways than current risk indicators.

An important challenge will be how to communicate information on small incremental risk increases conferred by emerging genetic markers for chronic disease risk. Currently the little empiric evidence available on these risk communications is confined to highly selected samples of well-educated patients for genes that confer high levels of risk. The increasing evidence base for common genetic polymorphisms that interact with common environmental risk factors to modestly increase chronic disease risk (e.g., *GSTM1* for smoking-related diseases, *PPARG* for diabetes, *COL1A1* for osteoporosis) offer research tools that can be used now to understand broader populations' response to genetic risk and to address other important public health questions (11).

Three decades of research in developing and testing behavior-change interventions for risk reduction tell us it is unlikely that a genetic test result alone will prompt behavior change. Yes, genetic test results might provide a cue to action to be capitalized upon and integrated with evidence-based multicomponent interventions already shown to influence behavior change. Moreover, consideration of who might be most interested in genetic testing and their motivations for such testing also could be explored to adapt intervention approaches accordingly.

## Reducing disparities in chronic disease outcomes

The prediction that genomic discovery may enable future population-risk stratification for chronic diseases raises understandable uneasiness about the use of genetic determinism to explain health disparities (12). This makes it all the more important that research now test how to use knowledge about the remarkable similarity of the human genome across time, continents, and populations to inform the discussion about what is social and what is biologic in our constructions of race, ethnicity, and other social groupings; such research could help us begin to clarify the individual and joint effects of these factors on chronic disease outcomes and health disparities. Scientists now suggest that at best, genetic predispositions may account for a third or less of chronic disease mortality (13). Communicating about the complex, probabilistic, and relatively weaker role of genetics in chronic disease could naturally open a dialogue about the stronger role of environment and, in turn, might be used to strengthen the potency of behavior-change interventions and to address the socially determined causes of disparities.

There are many fascinating and critical research questions about what are the most effective methods to increase the public's skills for evaluating the relative contribution of genetics to chronic disease outcomes, the fallibilities and strengths of genomics research, and to which groups these interventions should be targeted (e.g., racial or ethnic communities, patients, health care providers, health insurers, journalists, bench scientists). Moreover, how might these educational and skills-building interventions influence a target community's opinions and receptivity to emerging genomic discoveries and research?

Methodological research also should be a priority. For example, most of the large population genetics registries, despite earnest efforts, have had poor minority representation (14). From a scientific perspective, the external validity of study results based on these registries, as well as their credibility to minority communities, is lessened. Thus, it is critical now to evaluate different approaches to recruitment for genetic studies that augment minority and population-based recruitment. Moreover, exploring public education interventions to improve study recruitment could be a fruitful area of research. In this regard, research might also explore whether genetic susceptibility testing for chronic disease is viewed as a monolith, or whether participation in genomic research related to population-wide diseases (e.g., cancer, diabetes) and race- or ethnicity-associated diseases (e.g., cystic fibrosis, sickle cell anemia) are viewed differently by target groups.

Equal access to genomic technologies also will be important to reducing disparities in chronic disease outcomes. Again, getting started early will be critical if we are to design technologies that have any potential for dissemination (15). To this end, it is important to evaluate genetic testing and feedback in naturalistic settings such as public health clinics to better understand system barriers and facilitators that must be considered as we develop genomic technologies for broad-based dissemination (16). In each case, rigorous evaluation of delivery approaches that increase the likelihood that genomic technologies are accompanied by appropriate support services and are affordable to individuals and/or systems will be key to success.

## Improving chronic disease care effectiveness and efficiency

Interventions to help patients manage the physical and psychological consequences of their chronic conditions and make requisite lifestyle changes have shown benefits for a variety of patient and system-level outcomes (17). Yet effective self-management involves trial and error, as clinical recommendations are based on broad and heterogeneous phenotypes of chronic disease. An important question is whether genetically customized management recommendations could improve patient self-management of chronic illness above current standard-of-care approaches. For example, psychological theories tell us it is plausible and testable that genetically customized self-management interventions might empower patients to be better self-managers and consumers of health care. Accordingly, genetic tailoring might improve patient–provider relationships in ways that reduce visit time and follow-up needs. Additionally, we might ask what genetic information patients and providers need to make them better collaborators. Answers to these questions "upstream" might be used to direct bench science to genomics research where products have the best potential for dissemination to these target groups.

Also important to consider is that health care providers are expected to deliver an increasing number of preventive services during their visits with patients (18). Thus, the potential for genomic risk stratification to enable efficiencies in health care delivery that reduce cost without compromising care is an important area for research. Research related to current genetic testing applications (e.g., *BRCA1*, *BRCA2*, hereditary nonpolyposis colorectal cancer [HNPCC]) has been conducted in specialized care settings where certified genetic counselors provide one- to three-hour sessions to support patient decision making and communicate test results. This research tells us little about how these applications might be incorporated into primary care or community health settings. Evaluating different counseling delivery models that have been shown in previous health promotion research to be effective (e.g., lay advisors, telephone counseling, Web-based information) is a good place to start. This evaluation will require us to involve genetic counselors to balance what is best practice for communicating about chronic disease markers against what can be effectively integrated into a variety of care settings.

Cost also will be important to consider. Current studies have shown some pharmacogenomic interventions to be worth the cost, but these studies are too few in number to evaluate the implications of genomic medicine broadly (19). The importance of research for evaluating the interventions that might be most cost-effective upstream of genomic technology development cannot be overstated (19).

## What do we need to move forward a public health research agenda?

Special journal editions like this one and the research that is highlighted is a good start. Bringing the theme of genomics to national public health and behavioral medicine meetings and featuring public health scientists in the vanguard of this research from the Public Health Genomics Centers, funded by the Centers for Disease Control and Prevention, and the Centers of Excellence for ELSI Research, funded by the National Institutes of Health, as keynote speakers also could increase buy-in.

The slower pace of genomic discovery in chronic disease means that for the time being we will be using imperfect genomic-risk prototypes (20). Certainly, we must have standards for choosing which prototypes to evaluate (e.g., meta-analyses as an evidence base) but not hold them now to standards such as clinical validity or utility that ultimately may be the goal for dissemination. Indeed, why put the cart before the horse if the technologies in their prototypic form cannot accomplish goals that will affect public health outcomes?  Genome scientists, clinicians, and public health researchers could collaborate in developing working standards for selecting promising genomic-risk applications to be used in chronic disease research. It will be important to secure buy-in for this research from institutional review boards that may be uncomfortable with the use of experimental genomic technologies in public health and clinical settings.

Compromising on prototypes does not mean that our research should compromise on rigor. It is time to move beyond descriptive and exploratory studies to conceptually based, hypothesis-driven public health research. Public health researchers have a trailblazing role to play in these earliest phases of framing an agenda for genomics research that puts public health challenges front and center. The time for action is now!
